# Supplementary material for: The FLOWERING LOCUS T LIKE 2-1 gene of Chenopodium triggers precocious flowering in Arabidopsis seedlings
Source: Plant Signal Behav. 2023 Jul 28;18(1):2239420. doi: 10.1080/15592324.2023.2239420 (PMC10392752; doi:10.1080/15592324.2023.2239420)
Supplement: Supplemental Material [file KPSB_A_2239420_SM4704.docx]

***Table S1*.** Primers used in qPCR and for amplification and domestication of *FTL*

genes Primers used in qPCR (in 5'->3' orientation)

| Gene | Primer Name | Sequence | Annealing temperature |
| --- | --- | --- | --- |
| *AtUBQ10* | AtUBQ10 _F | GAAGTTCAATGTTTCGTTTCATGT | 58 °C |
|  | AtUBQ10 _R | GGATTATACAAGGCCCCAAAA |  |
| *CfFTL1* | HM_FTL1_111For | ATGAGAACCCAAGACCAGCA | 60 °C |
|  | HM_FT_R1 | CTCCCTCCCTCTGGCAAT |  |
| *CfFTL2-1* | HM_FTL2-1_111F | GGTGGTTTGTTATGAGAGCCC | 60 °C |
|  | HM_FTL2-1_111R | TAGTGATGAAATTCGGGCGC |  |
| *CfFTL2-2* | HM_FTL2-2_31F | AGGGAATTCTGAAGCATCCT | 60 °C |
|  | HM_FTL2-2_311R | TCCACCCAAATCTCCTTCCC |  |
| Basta | BAR_F | CTACATCGAGACAAGCACGGT | 58 °C |
|  | BAR_R | CTGAAGTCCAGCTGCCAGAA |  |

Primers used for amplification and domestication of the *FTL* genes from *C. ficifolium* and *C. quinoa*

| Gene | Primer Name | Sequence |
| --- | --- | --- |
| *CfFTL1* | GB-CfFT1_F | GCGCCGTCTCGCTCGAATGCCTAGAGCACCACCAAG |
|  | GB_CfFT1_R_S | GCGCCGTCTCGCTCAAAGCTTACAACCTTCTTCCACCAG |
|  | GB-CfFTL2-1_F | GCGCCGTCTCGCTCGAATGCCTAGAACAGCTTCAACA |
| *CfFTL2-1* | GB_CfFTL2-1_R_S2 | GCGCCGTCTCGCTCAAAGCTTACAACCTTCTTCCACCACAACCTCCTTCC CT |
| *CfFTL2-2* | GB-CfFTL2-2_F | GCGCCGTCTCGCTCGAATGGATTCCAGAGAAGAACTC |
|  | GB_CfFTL2-2_R_S | GCGCCGTCTCGCTCAAAGCTCACGAGCTTAATATAATGTGGA |

*CqFTL2-1* GB-CfFTL2-1F GCGCCGTCTCGCTCGAATGCCTAGAACAGCTTCAACA GB-CqFTL2-1R-S GCGCCGTCTCGCTCAAAGCTTACAACCTCCTTCCACCA

***Table S2***. DNA components used for the construction of plasmids for permanent transformation of Arabidopsis.

| **Assembly** | **Plasmid ID** | **DNA part** | **Length** | **Source** | **Original**  **reference** |
| --- | --- | --- | --- | --- | --- |
| alpha11 | pUPD1-  CsVMV A1B2 | CsVMV Promotor | 538 | this work, Virology  lab, IEB | Verdaguer  *et al.,* 1998 |
| alpha11 | pUPD1-VGE  B35 | VGE chimeric  transcription factor | 1752 | this work, Virology  lab, IEB | Semenyuk  et al., 2010 |
| alpha11 | pICH44300 | *A.thaliana* actin2  terminator | 485 | MoClo kit | Engler *et al*.,  2014 |

| alpha12 | pUPD1 TM2-  MAR | *N.tabaccum*, MAR | 1001 | Virology lab, IEB | Dušek *et al*.,  2020 |
| --- | --- | --- | --- | --- | --- |
| alpha13 | pUPD1- 5xM35S  A1B1 | inducible promotor | 172 | this work, Virology lab, IEB | Semenyuk  *et a*l., 2010 |
| alpha13 | pUPD1-TMV-  Omega B2 | transcriptional enhancer from Tobacco mosaic  virus | 76 | this work, Virology lab, IEB | Sleat *at al*., 1987 |
| alpha13 | pUPD2-Ftl-2-  1-B35 | *FTL2-1* CDS | 549 | this work, Plant  Reproduction lab, IEB | Štorchová *et*  *al.* 2019 |
| alpha13 | pICH41414 | CaMV *35S* terminator | 204 | MoClo kit | Engler *et a*l.,  2014 |
| alpha14 | pUPD1-Sf35 | short DNA stuffer  fragment | 45 | this work, Virology  lab, IEB |  |
| omega1 | pUPD1 RB7 –  MAR | *N.tabaccum*, MAR | 1168 | Virology lab, IEB | Dušek *et al*.,  2020 |
| alpha1 | pP35S (GB0030) | CaMV *35S* promoter with TEV transcriptional  enhancer | 738 | this work, Virology lab, IEB, from pTF101 | Paz *et al*., 2004 |
| alpha1 | pUPD1 Basta | Bar, phosphinothricin  acetyltransferase | 561 | this work, Virology  lab IEB, from pTF101 | Paz *et al*.,  2004 |
| alpha1 | pUPD1 VTSP | *Glycine max*, Vegetative tissue storage protein  terminator | 545 | this work, Virology lab, IEB, from pTF101 | Paz *et al.,*  2004 |
| alpha2 | pUPD2-  OleFast | RFP protein fused to *A.*  *thaliana* oleosin CDS and promoter | 2311 | this work, Pollen lab,  IEB, adapted from MoCloKit | Engler *et al*., 2014 |

**Dušek J, Plchová H, Čeřovská N** *et al***. 2020.** Extended Set of GoldenBraid Compatible Vectors for Fast Assembly of Multigenic Constructs and Their Use to Create Geminiviral Expression Vectors. *Frontiers in Plant Sciences* **11:** 522059

**Engler C, Youles M, Gruetzner R, Ehnert TM, Werner S, Jones JD, Patron NJ, Marillonnet S. 2014.** A golden gate modular cloning toolbox for plants*. ACS Synthetic Biology* **3**: 839-843.

**Paz MM, H. X. Shou HX, Guo ZB, Zhang ZY, Banerjee AK, Wang K. 2004.** Assessment of conditions affecting Agrobacterium-mediated soybean transformation using the cotyledonary node explant. *Euphytica* **136**: 167-179.

**Semenyuk EG, Schmidt MA, Beachy RN, Moravec T, Woodford-Thomas T. 2010.** Adaptation of an ecdysone-based genetic switch for transgene expression in soybean seeds. *Transgenic Research* **19:** 987-999.

**Sleat DE, DGallie DR, Jefferson RA, Bevan MW, Turner PC, Wilson TM. 1987.** Characterisation of the 5'- leader sequence of tobacco mosaic virus RNA as a general enhancer of translation in vitro. *Gene* **60**: 217-225.

**Štorchová H, Hubáčková H, Abeyawardana OAJ, Walterová J, Vondráková Z, Eliášová K, Mandák B. 2019.** *Chenopodium ficifolium* flowers under long days without upregulation of *FLOWERING LOCUS T (FT)* homologs. *Planta* **250:** 2111-2125.

**Verdaguer B, de Kochko A, Fux CI, R. Beachy RN, Fauquet C 1998**. Functional organization of the cassava vein mosaic virus (CsVMV) promoter. *Plant Molecular Biology* **37**: 1055-1067.
